# Open synthetic travel demand for Paris and Île-de-France: Inputs and output data

**DOI:** 10.1016/j.dib.2021.107622

**Published:** 2021-11-23

**Authors:** Sebastian Hörl, Milos Balac

**Affiliations:** a Institut de Recherche Technologique SystemX, Palaiseau F-91120, France; b Institute for Transport Planning and Systems, ETH Zurich, Zurich CH-8092, Switzerland

**Keywords:** Synthetic, Population, Travel demand, Transport simulation, Paris

## Abstract

Hörl and Balac [1] describe a data pipeline to generate a synthetic travel demand for Paris and Île-de-France. The data set consists of households, persons and their daily activity chains. It can be used in transport simulation, energy analysis and other research fields such as epidemiology. This data-in-brief article describes in detail the generated data set and how it can be regenerated based on publicly available and open data. The characteristics and pre-processing steps for the input data sets are covered in detail.

## Specifications Table

**Table:** 

Subject	Data engineering
Specific subject area	Population and travel demand synthesis
Type of data	Database of households, persons and daily activity chains
How data were acquired	A range of open and publicly available data sets are cleaned, processed and fused.
Data format	Secondary data
Parameters for data collection	The used input data is open and publicly available. It is provided and maintained by French authorities, ensuring long-term access and reproducibility.
Description of data collection	All referenced data sets can be downloaded from the French authorities. Links are provided throughout the paper. This process can be repeated any time.
Data source location	Primary data sources: French population census 2015 (INSEE) [7], [8], [9], [10]
	National tax database 2015 (INSEE) [11]
	National service and facility database 2020 (INSEE) [14]
	National Household Travel Survey 2010 (INSEE) [6]
	National enterprise census (INSEE) [12]
	Statistical zoning system 2017 (IGN) [4], [13]
	National address database December 2020 (IGN) [3]
Data accessibility	Repository name: Mendeley Data Data identification number: 10.17632/p3v8zmps2w.1 Direct URL to data: https://doi.org/10.17632/p3v8zmps2w.1
Related research article	S. Hörl, M. Balac, Synthetic population and travel demand for paris and Île-de-france based on open and publicly available data, Transportation Research Part C: Emerging Technologies 130 (2021) 103291. https://doi.org/10.1016/j.trc.2021.103291

## Value of the Data

• Synthetic population and synthetic travel demands are an important basis for simulations and analyses in a range of fields such as transport, energy, and land use.
• The data can benefit users and developers of simulation tools in transport, land use and energy analysis.
• As a benchmark, it will support the development and testing of advanced population synthesis algorithms and transport simulation tools.

## 1 Data Description

In [1] the authors describe a comprehensive pipeline from raw input data sets to a final data set representing the travel demand for the Île-de-France region around Paris. While additional details on the collection and pre-processing of input data is given further below, the output data, which has been made publicly available [2], is described in the following.

The output data of the travel demand synthesis is structured as follows:

• meta.json contains metadata, including with which random seed or sampling rate the population was created and the date and time of creation.
• persons.csv and households.csv contain all persons and households in the population with their respective sociodemographic attributes.
• activities.csv and trips.csv contain all activities and trips in the daily mobility patterns of these people including attributes on the purposes of activities or transport modes for the trips.
• activities.gpkg and trips.gpkg represent the same trips and activities as spatial data in *GeoPackage* format that can be processed in geographical information systems. Activities contain point geometries to indicate where they happen, and the trips file contains line geometries to indicate the origin and destination of each trip. The coordinates are given in EPSG:2154 projection which is commonly used in France.
• homes.gpkg contains all household locations referencing the household data set from above.

The household data set contains the following attributes per household: household_id as a unique identifier; car_availability (all / some / none) indicating if all, some, or none of the persons in the household have access to a car; bike_availability (all / some / none) indicating if persons have access to a bicycle; income describing the total monthly household income; and census_household_id as the identifier of the household from the raw census input which was used to generate the present synthetic household.

The **person** data set contains the following attributes per person: person_id as a unique identifier; household_id referencing the household the person is living in; age of the person in years; employed indicating whether the person is currently employed; sex (male / female) indicating sex of the person based on census data; socioprofessional_class (1 - 8) following the French defininition of socioprofessional classes [5]; has_driving_license (bool) indicating whether the person has a driving license; has_pt_subscription indicating whether the person has a public transport subscription; census_person_id as the identifier of the person from the raw census input which was used to generate the present synthetic person; and hts_person_id as the identifier of the person from the household travel survey which was used to create the synthetic person.

The **activity** data sets contain the following attributes per activity: the person_id of the person whose activity chain the activity is part of; the household_id of that person; an activity_index (0 - N) indicating the location of the activity in the acitivity chain; preceding_trip_index and following_trip_index indicating the index of the preceding and following trip in the activity chain (see below) for easier analysis and referencing; the purpose of the activity (home / work / education / shop / leisure / other); the start_time and end_time (in seconds) of the activity; and two boolean variables indicating whether the activity is_first or is_last (boolean) in the agent’s activity chain.

The **trip** data sets contain the following attributes per trip: the person_id of the person whose activity chain the trip is part of; the household_id of that person; an trip_index (0 - N) indicating the location of the trip in the activity chain; preceding_activity_index and following_activity_index indicating the index of the preceding and following trip in theactivity chain (see above) for easier analysis and referencing; the departure_time and arrival_time of the trip in seconds; the mode of transport for the trip (car / pt / bike / walk); preceding_purpose and following_purpose indicating the purpose of the preceding and following activity (see above); and whether the trip is_first or is_last (boolean) in the agent’s activity chain.

The data set provided along this article is a sample of 5% of the households in Île-de-France. While a full population is rarely needed and used in transport simulation (as it takes excessive time to perform the simulations), 5% is a value that is feasible and practical for a region as large as Île-de-France. Furthermore, the analyses of the population in the accompanying paper are performed on a 5% sample of the population. Since, given the publicly available, raw, input data sets, the pipeline is completely re-runnable by anyone, this does not pose a restriction, but larger sample sizes can be generated at any time by researchers.

## 2 Experimental Design, Materials and Methods

As described in [1], the synthesis pipeline aims to start with raw data sets, to transform them, to apply further models, and arrive at a final synthetic travel demand that can be readily used in a downstream agent-based transport simulation. We intentionally apply rather simple, data-driven algorithms to establish a baseline into which more sophisticated models can be integrated later on, with the ability to compare them against an established benchmark. The process outputs are determinstic given a configurable random seed, which was chosen to be 1234 for the output data set linked to this article.

All processing steps and their implementation in Python is available online at:

https://github.com/eqasim-org/ile-de-france

The specific version (v1.2.0) which has been used to generate the static data set linked to this article is available at:

https://github.com/eqasim-org/ile-de-france/tree/v1.2.0

Unfortunately, the distribution systems of the external data providers have changed since release of v1.2.0, so researchers interested in running the pipeline on their own are kindly referred to the most recent version of the pipeline at any time. The repository provides instructions that make it possible for anybody with basic knowledge of Python to create a synthetic travel demand as discussed above. Three steps need to be followed:

• Collection of data sets: We do not provide the output data of the pipeline, but anybody can regenerate it. For that, the individual raw source data sets need to be collected. The repository gives instructions on where to find the data sets, how to download them, and how to arrange them as input data for the pipeline. All of them are publicly accessible.
• Running travel demand synthesis: Second, instructions are provided on how to run the demand synthesis. For that, *Python* needs to be installed. We explain how to install all needed dependencies in a *conda*^1^ environment, how to apply minimal necessary adjustments to the pipeline configuration (setting the path to the input data), and running the code. The output is the travel demand data, as described above.

The following sections give an overview of the input data sets and how they are cleaned. The process of fusing them is described in the main article [1].

### 2.1 Spatial zoning system

In France, different spatial zoning systems are in use for statistical purposes. In this research, only a couple of them is used, mainly the ones that have the highest availability among the published data sets. The reference data set is a shapefile containing the contours of all IRIS zones in France [4], [13]. Those IRIS zones were introduced in 1999 and updated in 2008 to perform country-wide statistical analyses based on the national census. Each IRIS zone has an identifier that is divided into three parts: The first two digits denote the *département*, which is an upper-level administrational unit, the following three digits identify a *commune* (municipality) within this *département*, and the last four digits describe the statistical IRIS zone in that municipality. It is important to note that not all *communes* may be divided into IRIS, mainly if they have less than 10,000 inhabitants.

**Fig. 1:**
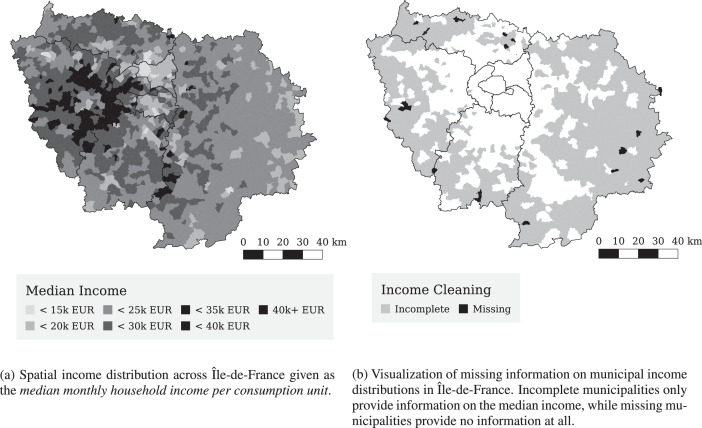
Analysis of household income distributions in Île-de-France, based on oepn data from the national tax data base [11].

For the present study, only zones within the Île-de-France *region* in France are considered. Those are all which lie in the departments of Paris (75), Seine-et-Marne (77), Yvelines (78), Essonne (91), Hauts-de-Seine (92), Seine-Saint-Denis (93), Val-de-Marne (94), and Val-d’Oise (95). Fig. 1a in the main article [1] shows the area of the *départements* of Île-de-France and the *communes* into which they are divided. The city area of Paris is not divided into *communes*, but into 20 *arrondissements*. Their spatial extents are similar to those of the municipalities. Therefore, both *communes* and *arrondissements* are treated equally in the scope of our method. The segmentation of the Paris department into *arrondissements* is shown in Fig. 1b in the main article [1]. In this case, also the further division into statistical IRIS zones is shown.

**Table 1:** Attributes per person resulting from cleaning and analysis of the French census dataset.

Structural attributes	Person attributes	Household attributes	Spatial attributes
Household ID	Age	Household size	IRIS ID*
Person ID	Sex	Number of cars	Municipality ID*
Household weight	Employed (yes/no)		Department ID
	Ongoing education (yes/no)		
	Socio-professional category		

*Spatial attributes are only given if available.

For the Île-de-France region, we work with eight departments, 1296 municipalities, and 5259 IRIS covering an area of around $12,000\;{\text{km}}^{2}$.

### 2.2 National census

National census data for France (*Recensement de la population*, RP) is published by *INSEE* (National Institute of Statistics and Economic Studies) on an annual basis three years after that data has been obtained [7]. The latest available data set has been published in June 2018 and contains statistical information of a representative sample of the French population for the year 2015. For each household and each person, numerous sociodemographic attributes are given, such as age, gender, household income, number of cars, and others. A statistical weight is assigned to each household, which makes the data compatible with previous publications of the data set, and other surveys performed in France.

For most households, the identifier of their home IRIS is given, which allows for the protection of person-specific data, but makes it also possible to use the data for synthesis purposes, as in this study. If an IRIS has less than 200 inhabitants, only the identifier of the municipality is given (0.07% of weighted households). Also, some municipalities are not covered by IRIS at all, because the municipality itself has a low number of inhabitants. In those cases, only the identifier of the department is known (10.06% of weighted households).

Nevertheless, the national census allows us to synthesize a population with spatially detailed sociodemographic attributes. It is also fortunate that the census is given on the household-level, with specific persons being directly attached to those households. This structure makes it possible also to synthesize realistic household-level distributions of sociodemographic attributes.

The census data set for 2015 features a large sample of individually weighted person observations for France and its overseas territories. For the Île-de-France region, it lists around 4.3 million residents in 1.9 million households, which makes around 35% of the real population.

For its use in our approach, the individual dataset is further cleaned. The resulting attributes can be seen in Table 1. Households containing persons who have their principal place of employment or education outside of Île-de-France are filtered out to restrict the study area to the region itself. These are 1.97% of the households containing 2.53% of all persons.

One attribute that requires further explanation is the *socio-professional category*. It is a well-defined [5] concept which classifies persons into eight categories: (1) agricultural workers, (2) artisans, merchants, self-employed, (3) leading positions and intellectual workers, (4) intermediate professions, (5) employees, (6) workers, (7) retired, (8) others without employment.

In addition to the detailed data sets, INSEE also prepares aggregate datasets. These tables contain, for instance, an aggregated age distribution for each IRIS in France, including those whose inhabitants cannot be geolocalised in the individual data set. We use this dataset to enrich the zoning data with information on the total number of inhabitants in each zone. Furthermore, we use the data as a reference when comparing the characteristics of our synthetic population.

### 2.3 Origin-Destination commute flows

Along with the census data, information about the commuting behavior of the French population is published by INSEE on an annual basis [8], [9]. For most of the individuals in the census data set, the origin-destination (OD) pair for the daily commute is recorded.

The information is provided in two data sets: One for work commute and one for commuting to educational activities such as school or university. In each case, the origin *commune* is given, along with the destination *commune*. Furthermore, the commutes are annotated with a statistical weight and the used mode of transport. It is not possible to reconstruct the actual individuals of the census data from these commutes.

As can be seen from Fig. 1a in the main article [1], commuting flows between municipalities can be understood as quite detailed from the perspective of the whole Île-de-France region. In Paris, however, this refers to the *arrondissements*, which renders the data set quite sparse. The potential of the data set is, therefore, not to produce highly realistic commuting patterns on a lower level like within the center of Paris. Instead, we use it to model the overall movement of people in and out of the city.

For the municipalities within Île-de-France, the data set contains around 8.3 million observations for work commutes and around 43,000 observations for education commutes.

Two OD matrices are derived from the data set, one for commutes to work and one for commutes to education. The process is straightforward: For each origin municipality, the weighted number of trips to each other municipality (destination) is tracked and divided by the total number of originating trips. This way, a probability of commuting to a particular destination municipality is established for each origin municipality. In only four cases (one for work and three for education), no trips are recorded at all for a specific origin. In these few cases, only trips inside the same zone are allowed.

### 2.4 Income distribution

The Filosofi (*Fichier Localisé Social et Fiscal*) data set collects income data of tax registered people in France. It is published as open data three years after the acquisition of the data [11]. The most recent data set has been published in 2018 and therefore contains income information of the population in 2015. Specifically, the data set provides the centiles of the distribution of *declared income* and *disposable income*. While the former mainly considers gross salaries, the latter takes into account deductions due to taxes, social security, state insurances, as well as social benefits. The distributions are given on the level of regions and municipalities, but one year after (i.e., four years after acquiring the data), a more fine-grained data set is published that provides distributions on the level of IRIS. The latter data set, however, does not provide further sociodemographic information, while the municipality-based version also offers income distributions by household size and a few other household-level sociodemographic attributes.

In the synthesis process, we use the regional Filosofi data set for validation. For synthesis, we only make use of the general income distributions by municipality. Further sociodemographic information could be included in the future.

The income provided in Filosofi is an annual income *per consumption unit*. It is a metric that makes incomes comparable between households. Since different household configurations entail different consumption patterns, the total income is not directly divided by the number of household members, but each of them is assigned a specific weight. INSEE, therefore, defines a consumption unit (*unité de consommation*) such that the first person over 14 years is counted as one full unit. Then, every additional person over 14 years is weighted by 0.5, while every person under 14 is weighted as 0.3. These definitions are important to arrive at meaningful comparisons of income distributions of different data sets. Income classes, for instance, in the household travel surveys (see below), usually refer to the *monthly disposable income per consumption unit*.

The distributions are given in eight centiles from the 10% to the 90% centile. Unfortunately, not all municipalities (715 of 1,296) in Île-de-France provide all centiles, mainly due to data protection for those areas with small population density. For these cases, only the median income in the municipality is known. Furthermore, 19 municipalities are not contained at all in the tax data set.

To clean the data, we first impute an income distribution to all municipalities for which only the median is known. We do so by comparing the median income values of the incomplete municipalities with the median income values of all known distributions. We then select the known municipality with the closest median and attach the full distribution to the incomplete municipality. In the future, more detailed imputation procedures could be applied, e.g., an additional matching by the Gini coefficient. Second, we fix municipalities that are missing altogether by finding the nearest (centroid) neighbor municipality of each of them. We then attach the income distribution of the neighbor municipality to the missing one.

Fig. 1a shows the spatial distribution of median income per municipality. The median household income varies between around 13,000 EUR and 43,000 EUR across all municipalities. The overall median in Île-de-France (derived from the regional data set) is around 23,000 EUR. From Fig. 1a one can see how household incomes are relatively higher in the west of Paris while incomes in the eastern suburbs are substantially lower. Fig. 1b shows all municipalities for which some cleaning procedure was necessary. One can see that completely missing data is very rare, while municipalities with incomplete income distributions are located relatively far away from the city center of Paris. Especially, Paris itself and the three surrounding *départements* are covered well with detailed income distributions.

### 2.5 Household travel survey

The *Enquête nationale transports et déplacements* (ENTD) is the national household travel survey (HTS) for France conducted between April 2007 and April 2008. While the data is rather old, it still provides valuable information on daily mobility patterns [6].

The data set is divided into several parts that are relevant for the study at hand: For each household and each person within, detailed sociodemographic information is available such as age and gender. Income classes by household are also available. Furthermore, one particular day is described in detail for the household’s reference person by a chain of trips. Each trip holds information about the preceding and following activity, the mode of transport, distance, and duration. In terms of spatial information, the data is relatively coarse since the trip start, and end locations are only given on the level of departments. For the study at hand, the data set is merely a rich source of information on the daily patterns of individual travelers. Since sociodemographic attributes are given, a connection to the census data can be established. Given a set of artificial agents with attributes such as age and gender, it is, therefore, possible to find activity chains with similar sociodemographics and attach them to those agents. Furthermore, the ENTD gives a rich set of reference distributions, such as departure and arrival times during one day by mode of transport, mode shares in general or by the time of day, distances covered, and more. It, therefore, provides information that can help to validate the behavior of the synthetic population. In terms of income, the ENTD provides household income classes per consumption unit.

The ENTD contains 20,200 households, among which 5823 households with 14,216 persons are located in the Île-de-France region. Only 4,613 respondents are surveyed about their daily mobility patterns, which resembles 0.04% of the population of Île-de-France. It should be noted that for the region also the regional travel survey *Enquête globale de transport* (EGT) [16] is available on request from the authorities and that the methodology presented below can be used equally with this data source. While it provides more recent (2010) and detailed data (35,000 respondents in 15,000 households with detailed trip chains for *all* household members), the sparser ENTD is used here to provide an entirely reproducible synthetic travel demand.

Inside the synthesis pipeline, the ENTD is prepared as follows. Table 2 summarizes the extracted attributes. Those attributes written in *italic* have the same set of possible values as the census data described above and can, therefore, be used for matching trips and activity chains to artificial persons. The *income class* is defined by income strata, which, however, does not impose any restriction on the matching process, as will be shown further below. Additionally, the consumption units per household are calculated as defined previously.

**Table 2:** Attributes resulting from cleaning and analysis of the French HTS datasets. Attributes in *italic* have the same structure as the census attributes in Table 1.

Structural attributes	Person attributes	Household attributes
Chain ID	*Age*	*Département ID*
	*Sex*	*Household size*
	*Employed (yes/no)*	*Number of cars*
	*Ongoing education (yes/no)*	Consumption units
	*Socioprofessional category*	Income class*
	Driving license (yes/no)	Number of bicycles
	Public transport subscription (yes/no)	

*Defined as income strata.

**Fig. 2:**
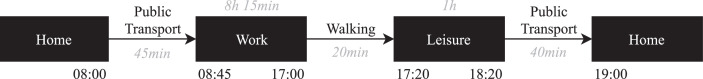
Example of an activity chain with available attributes from French HTS data. Derived attributes are shown in light gray.

On the trip level, the ENTD is cleaned to provide the following attributes: departure time, arrival time, the purpose of the following activity, the purpose of the preceding activity, mode of transport, origin department, destination department, and (Euclidean) distance. Fig. 2 summarizes the available information plus additional information such as trip and activity durations, which can be easily derived from the data. We consider *car driver, car passenger, public transport, bicycle*, and *walk* as modes of transport. While the HTS allows for a more detailed analysis, those are the ones that we consider essential for a first version of the model that can be refined later on. For activities, we use the specific types of *home, work, education* (which will be referred to as *primary activities* in the following), and *leisure, shopping*, and *other* (*secondary activities*). The same concept applies as for the modes of transport: A much more fine-grained distinction would be possible later on, but is not considered in the basic model. For instance, introducing a distinct *food* category for trips to a restaurant or lunch break could be a straightforward extension.

Finally, the ENTD is cleaned to reference the group of people staying in Île-de-France: Persons with trips that go beyond the border of Île-de-France are deleted from the data set, as well as those which do not have their residence in the area.

### 2.6 Address database

The National geographic institute of France (IGN) provides a regularly updated and publicly accessible database of all registered addresses in France called BD-TOPO [3]. It contains the written address including street name and house number, as well as a distinct coordinate for each observation. For Île-de-France, the data set contains 2,131,728 individual addresses, of which 1,891,175 can be used in our process because they have valid street names, house numbers and municipality identifiers. The data set allows us to define locations of daily activities in a detailed way.

#### 2.6.1 Enterprise census

An open and publicly accessible enterprise census exists (SIRENE, *Systéme national d’identification et du répertoire des entreprises et de leurs établissements*), which lists all enterprises registered in France. It further divides enterprises into individual facilities with unique identifiers. For each enterprise and facility it provides the number of employees and the type of sector according to the official enterprise classification system of France (NAF, *Nomenclature d’activités française*). While the data set provides the address of each facility in written form, as well as the identifier of their municipality, their location is not known by coordinate in an easily digitally processable way.

Therefore, we match the 411,608 available facilities in Île-de-France with the address database. 379,175 of addresses can be matched exactly to the coordinate by using street name, house number and municipality identifier. 7521 can be matched without taking the municipality into account (to fix cases in which the address database and the enterprise database may not be synchronized due to municipality mergers or separations). Finally, an additional 5467 are matched by municipality identifier and a Levenshtein distance [15] compared to a candidate from the address database of at most five modifications. In total, that gives 392,163 (95.28%) enterprises that can be geolocalized on three different levels of confidence.

The enterprise census data is used to define work places for the synthetic population in detail.

For the SIRENE data set, it must be mentioned that the data is updated every month without direct access to previous versions. While the processing pipeline can be always run with the most recent version, recovery of the exact output data set linked to this article is, unfortunately, not possible without access to the specific SIRENE data set from *September 2020*. While such a component is not implemented in the pipeline today, the data set at any point in time *could* be reconstructed exactly since detailed change set data (creations, deletions) for all monthly updates are available. Such a commponent is planned for future iterations of the pipeline code.

#### 2.6.2 Service and facility database

A service and facility census (BPE, *Base permante des équipements*) is published on an annual basis by INSEE [14]. It consolidates several independent data sets with the goal of establishing a central registry that lists services and facilities with their location and type in France. While many are annotated with exact coordinates, some are only known by IRIS or municipality.

During the clean-up process, all observations are deleted, which do not provide either a valid IRIS or municipality identifier within Île-de-France. All remaining observations that do not provide exact coordinates are placed at a random address (see above) inside their respective IRIS if it is specified, otherwise inside their associated municipality. After applying this process, we arrive at 469,181 facilities.

The cleaned *BPE* allows us to assign the location of agent activities such as shopping realistically during demand synthesis. Analogously to the activity types mentioned above, we divide the facility type into four categories: *education* (11,267 obs.), *shop* (67,458 obs.), *leisure* (64,416 obs.), and *other* (326,040 obs.). In theory, also the *BPE* would allow for a much more fine-grained definition of activity types, which offers the potential to improve the synthesis process in the future. The category *other* currently mainly consists of facilities from the sectors of health, transport, and tourism.

## Ethics Statement

The authors declare that this work does not involve the use of human subjects, social media data, or experimentation with animals.

## CRediT Author Statement

**Sebastian Hörl:** Conceptualization, Data curation, Formal analysis, Investigation, Methodology, Resources, Software, Validation, Visualization, Writing – original draft; **Milos Balac:** Conceptualization, Funding acquisition, Investigation, Project administration, Resources, Validation, Writing – original draft.

## Declaration of Competing Interest

We would like to acknowledge *Airbus Urban Mobility GmbH* whose funding has supported the development of the synthetic travel demand of the Île-de-France region around Paris.

The authors declare that they have no known competing financial interests or personal relationships which have, or could be perceived to have, influenced the work reported in this article.

## Footnotes

1 https://www.anaconda.com/
